# Retard scolaire révélant une ectopie cristallinienne chez une enfant de 7 ans

**DOI:** 10.11604/pamj.2014.18.7.4232

**Published:** 2014-05-02

**Authors:** Rajae Derrar, Rajae Daoudi

**Affiliations:** 1 Université Mohammed V Souissi, Service d'ophtalmologie A Hôpital des spécialités CHU Rabat. Maroc

**Keywords:** Retard scolaire, ectopie cristallinienne, syndrome de marfan, school retardation, ectopia lentis, marfan syndrome

## Image en medicine

**Figure 1:**
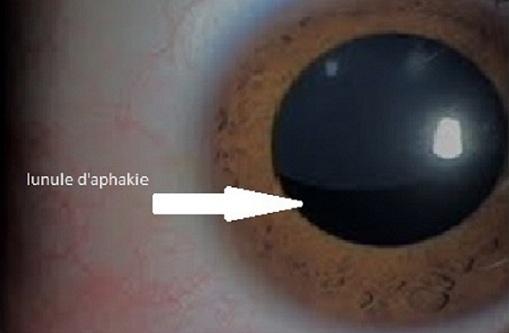
Ectopie cristallinienne

Enfant âgé de 7 ans issu d'un mariage consanguin présentant des difficultés à suivre en début de scolarité. L'examen général note sa grande taille par rapport à son âge avec maigreur et longs doigts fins. A l'examen on trouve une acuité visuelle limitée à 1/10, un bon réflexe photomoteur, l'examen du segment antérieur trouve une cornée claire avec chambre antérieure augmenté de profondeur et ectopie cristallinienne en supérotemporal (Figure 1) l'examen du fond d’œil trouve une papille d'excavation physiologique avec un bon reflet fovéolaire et l'absence de lésions dégénératives en périphérie. Un bilan étiologique a été demandé comprenant, un avis pédiatrique, un examen cardiovasculaire, et une échographie cardiaque qui ne notent pas de prolapsus mitral ou d'anévrysme aortique. L’électrophorèse des acides aminés dans les urines à la recherche d'une homocystinurie était négative. L'ectopie cristallinienne est un déplacement congénital du cristallin lié à une anomalie zonulaire. C'est une anomalie évolutive pouvant être isolée ou rentrer dans le cadre d'une maladie générale tel que (le syndrome de Marfan, l'Homocystinurie ou le syndrome de Weil Marchesani). Dans notre cas, devant l'aspect morphologique du patient et son ectopie cristallinienne, un syndrome de marfan a été fortement suspecté. Le patient a bénéficié d'une phacophagie avec correction de l'aphakie par des lunettes et suivie en consultation de strabologie afin de prévenir l'amblyopie.

